# Predicting Extreme Droughts in Savannah Africa: A Comparison of Proxy and Direct Measures in Detecting Biomass Fluctuations, Trends and Their Causes

**DOI:** 10.1371/journal.pone.0136516

**Published:** 2015-08-28

**Authors:** David Western, Victor N. Mose, Jeffrey Worden, David Maitumo

**Affiliations:** African Conservation Centre, 15289 00509, Nairobi, Kenya; Fudan University, CHINA

## Abstract

We monitored pasture biomass on 20 permanent plots over 35 years to gauge the reliability of rainfall and NDVI as proxy measures of forage shortfalls in a savannah ecosystem. Both proxies are reliable indicators of pasture biomass at the onset of dry periods but fail to predict shortfalls in prolonged dry spells. In contrast, grazing pressure predicts pasture deficits with a high degree of accuracy. Large herbivores play a primary role in determining the severity of pasture deficits and variation across habitats. Grazing pressure also explains oscillations in plant biomass unrelated to rainfall. Plant biomass has declined steadily and biomass per unit of rainfall has fallen by a third, corresponding to a doubling in grazing intensity over the study period. The rising probability of forage deficits fits local pastoral perceptions of an increasing frequency of extreme shortfalls. The decline in forage is linked to sedentarization, range loss and herbivore compression into drought refuges, rather than climate change. The results show that the decline in rangeland productivity and increasing frequency of pasture shortfalls can be ameliorated by better husbandry practices and reinforces the need for ground monitoring to complement remote sensing in forecasting pasture shortfalls.

## Introduction

Drought refers to a prolonged rainfall shortage and its impact on climate, hydrology, ecology and agriculture [1–3]. Rain-fed farmers and pastoralists in the rangelands that span a quarter of the earth’s land surface are especially vulnerable to food shortages caused by drought [3]. Over the last century extreme famines caused by droughts have declined around the world as subsistence farmers and herders have entered market economies and become less susceptible to vagaries of climate [4]. Africa is a stark exception because of its large number of subsistence communities and rapidly growing populations still dependent on rain-fed food production [5]. Examples of severe famines caused by droughts in Africa include the Sahel in the 1970s, Ethiopia in the 1980s and the Horn of Africa in the 1990s and 2000s. Extreme droughts still cause widespread starvation, malnutrition, high infant death rates and social disruption in these regions, often made worse by armed conflict and marginalization [4, 6].

Predictive science has become more important and accurate in forecasting droughts as populations have grown and the disruptive effects of famine have deepened. Early predictions, such as the Palmer’s Drought Index [7], used rainfall, temperature and other climatic variables as proxy measures of drought. [8] describes the early development and application of such indices in gauging meteorological, hydrological, and agricultural droughts.

Climatic proxies alone fail to capture the many factors that affect plant growth and senescence. Additional physical and biological variables have been added to drought forecasting to improve accuracy [9]. Better meteorological and earth monitoring tools and methods have improved drought predictions and proxy measures [10, 11]. As a result, early warning systems have shifted from statistical predictions based on climatic records to complex biophysical models of weather and climatic patterns and measures of vegetation biomass based on satellite spectral imagery.

Spectral imagery is now widely used to infer green and dry biomass, vegetation condition and structure, soil degradation and other factors [12, 13]. The Normalized Difference Vegetation Index (NDVI) [14], which uses the difference between infrared and near-infrared reflectance to predict green biomass growth and senescence [15, 16], has become widely used in early warning systems such as the Famine Early Warning System Network [17]. NDVI is, however, least sensitive during prolonged droughts when measures of the temporal and spatial distribution of plant biomass are most important to farmers and herders.

A number of studies have attempted to correct the distortions and overcome the limitations of NDVI in drought-prone areas [18–20]. Some studies have calibrated spectral signatures against direct measures of biomass on the ground to improve its spatial and temporal resolution [21]. These efforts have come up against the biophysical limitations of spectrometry in dry seasonal environments, and the lack of direct long-term measurements of green and dry biomass on the ground against which to test and calibrate NDVI indices [21]. Consequently, whereas NDVI has proved useful in tracking seasonal growth and large scale shifts in green biomass, its reliability and resolution falls during droughts [22]. Fine-scale differences in pasture biomass and quality, often associated with key resource areas, also determine the survival and productivity of wild and domestic herbivores during droughts [23].

Drought predictions of pasture shortfalls must take into account many socioeconomic and environmental variables [24]. The spatial variability of total pasture biomass as well as greenness are important factors determining the distribution, movement and survival of wildlife and pastoral livestock populations. Herbivore movements also determine the offtake of pastures through the course of a season. Variations in offtake can far exceed variations in production due to rainfall. Notwithstanding the importance of climate in governing vegetation production and composition in dry environments [25], herbivory, especially in late season pastures [26], may have far more influence on determining the severity and intensity of drought [2].

A central challenge for effective early warning systems is uptake. Many early-warning systems do not reach farmers and herders, particularly among remote and marginalized communities. If they do, they are seldom absorbed and acted on [27]. In part this is because large scale early warning systems often run counter to the perceptions of farmers and herders at the fine scale of herd management, and so lack credence. Improving the uptake of early warning systems therefore hinges on improving the accuracy and resolution of predictions in the harshest of times at a scale that matches a herder’s foraging range. Uptake also depends on getting information into the hands of local herders in a form they can grasp, weigh and act on [28].

Here we look at the accuracy of proxy measures in predicting periods of extreme pasture shortfalls in the rangelands of East Africa where the severity and frequency of droughts is a growing concern [29], largely attributed to climate change [30]. We use 20 ground plots monitored every four to six weeks over 35 years in the Amboseli ecosystem of southern Kenya to assess how well measures of rainfall, NDVI and grazing pressure predict seasonal and long-term variability and trends in pasture biomass across a range of habitats used by large herbivores in the course of seasonal migrations. We look at the reliability of rainfall, NDVI and grazing pressure as proxy measures for predicting long-term fluctuations in green, dry and total biomass. In particular we look at how the proxy measures fare in predicting extreme periods of pasture shortfall, periodicity, persistence effects and falling productivity. Finally, we look at the causes of declining pasture production, the rising frequency of extreme shortfalls and consider the implications and applications of the findings.

If the causes of pasture fluctuation are climatic, then shortfalls will be largely stochastic in nature and lie outside the control of pastoralists. If, on the other hand, the causes of extreme shortfalls are due in large part to factors such as stocking rates and herding practices, then the causes of shortfalls will be more predictable and manageable.

## Study area and Methods

In this study we bring together remote sensing data, ground based measurements of rainfall and vegetation characteristics, as well as to look at the causes and dynamics of drought in an East African pastoral system studied for over four decades [31]. The Amboseli Ecosystem, stretching north from Kilimanjaro on the Kenya-Tanzania border, spans the 388 km^2^ Amboseli National Park and surrounding pastoral lands. The ecosystem is defined by the seasonal movements of large herbivores and covers approximately 8,500 km^2^. Temperature ranges from monthly highs in the mid-30s (February) to the mid-20s Celsius (July), with a mean annual rainfall of 350 mm [32]. Rainfall is bimodal, with short rains generally falling in November and December and long rains from March to May. Periods of below average rainfall are common. Further details of the ecosystem are provided elsewhere [31].

Wild and domestic herbivores move seasonally according to vegetation conditions and water availability [33]. Historically, domestic and wild herbivores congregate in the swamps at the base of Kilimanjaro during the dry season and migrate to the neighboring bushlands and plains during the rains [34]. Most permanent swamps outside Amboseli National Park have been converted to crop farming in the last three decades [35], and farms have expanded down the lower slopes of Kilimanjaro [36]. Rangeland fragmentation and the loss of key dry season grazing reserves have increased pressures on both livestock and wildlife. The gradual compression of herbivores into a smaller pasture area, coupled with the loss of flexibility due to sedentarization [37, 38] and land-use change, has led to a loss of herbivore production in the ecosystem [39].

**Fig 1:**
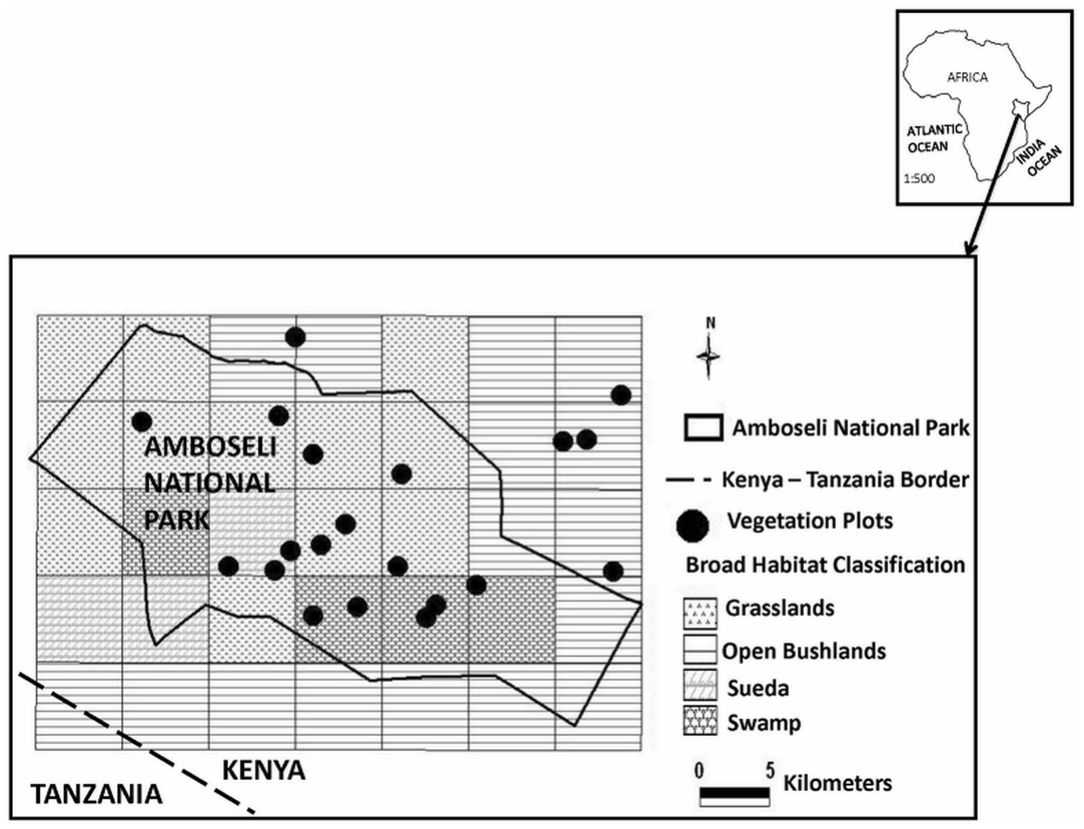
The distribution of 20 permanent vegetation plots across the habitats used by wildlife and pastoral livestock on seasonal migrations in the Amboseli ecosystem. The plots have been measured for standing crop biomass every 4 to 6 weeks since 1975. Sueda is gradually replacing the woodland habitat in the ecosystem.

Pasture conditions have been measured on 20 permanent plots every four to six weeks since 1975 [40]. The plots are spread across the major habitats in the Amboseli ecosystem: bushlands, grasslands, woodlands and swamps (Fig 1). Kenya Wildlife Service (KWS) issued permits to conduct field work inside the protected Amboseli National Park (latitude: -2.62661^0^; longitude: 37.254406^0^). For study sites located in the surrounding Olgulului group ranch (latitude: -2.605608^0^; longitude: 37.365875^0^) (Fig 1), no endangered plant species were involved. These plots were drawn from an initial sample of 110 randomized plots at the start of the monitoring. The variable estimates sampled fell within the means for each habitat, ensuring a representative sample of the Amboseli basin area. Biomass, relative greenness and grazing pressure are measured at each plot on a monthly basis using the point intercept method [41, 42]. For each frame, ten pins were dropped through the vegetation at an angle of 67^0^ [43]. Each plant hit was scored as green or brown and whether grazed or un-grazed. Total herbaceous biomass, total green, and total brown biomass was calculated using an equation derived from calibrating hits per pin against dry weight in grams per meter squared [40]. Grazing pressure was calculated as the percentage of grazed to non-grazed hits.

**Fig 2:**
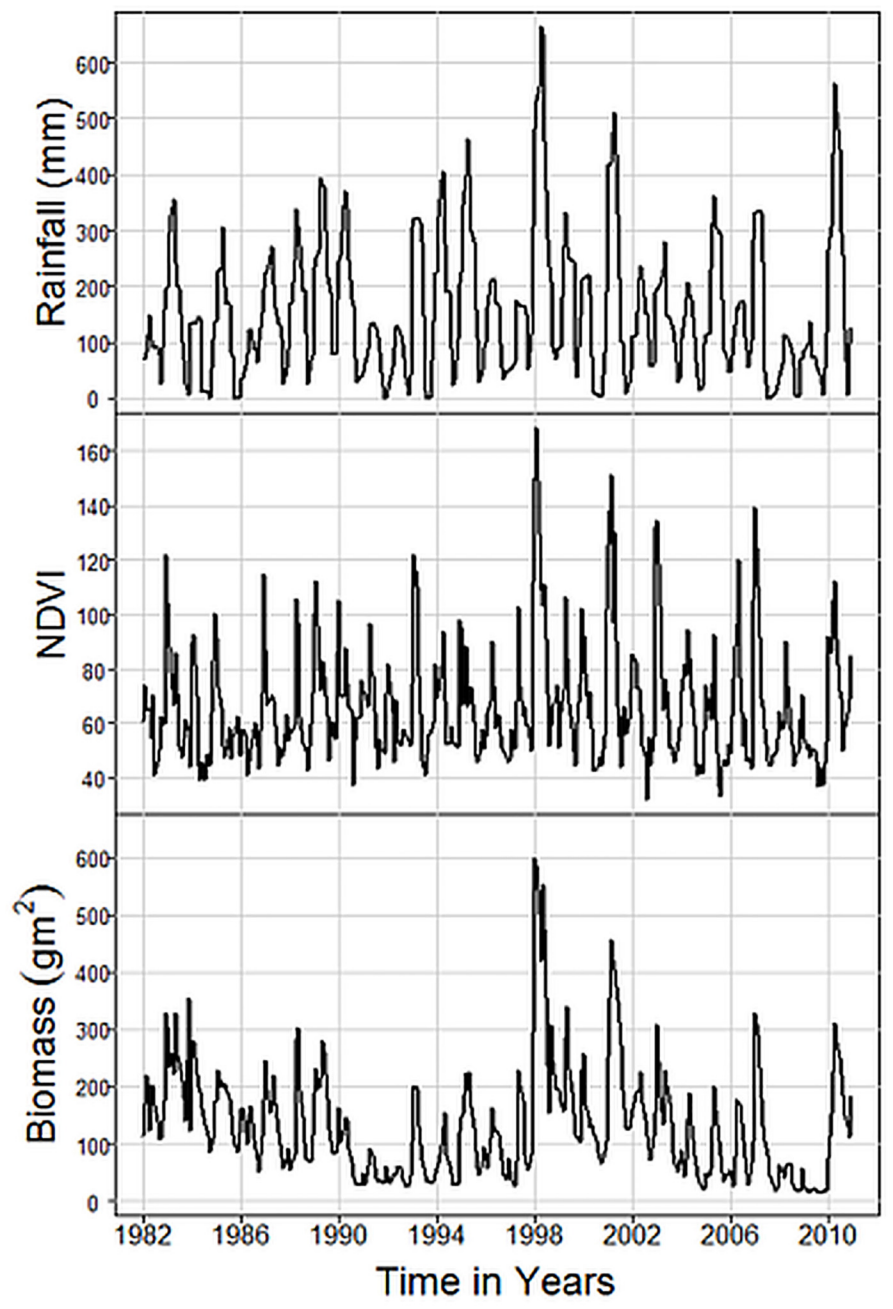
Monthly pasture biomass measurements aggregated for 20 plots, NDVI and rainfall over the period 1982 to 2010.

Measures of the Normalized Difference Vegetation Index (NDVI) were compiled from two different sources. For the period 1982 to 2006 we used NDVI images from AVHRR−GIMMS, obtained through the Global Land Cover Facility [44]. For the period 2006 to 2010, ASTER L1B data were obtained through the online Data Pool at the NASA Land Processes Distributed Active Archive Center (LP DAAC), USGS/Earth Resources Observation and the Science (EROS) Center, Sioux Falls, South Dakota [45]. We extracted NDVI values for each of the vegetation plot points at ten−day intervals and compiled a time series of maximum monthly NDVI. These data were then calibrated to obtain a uniform scale time series for each plot. Plot level values were averaged to create an aggregate NDVI value for the major habitats (Fig 2).

**Fig 3:**
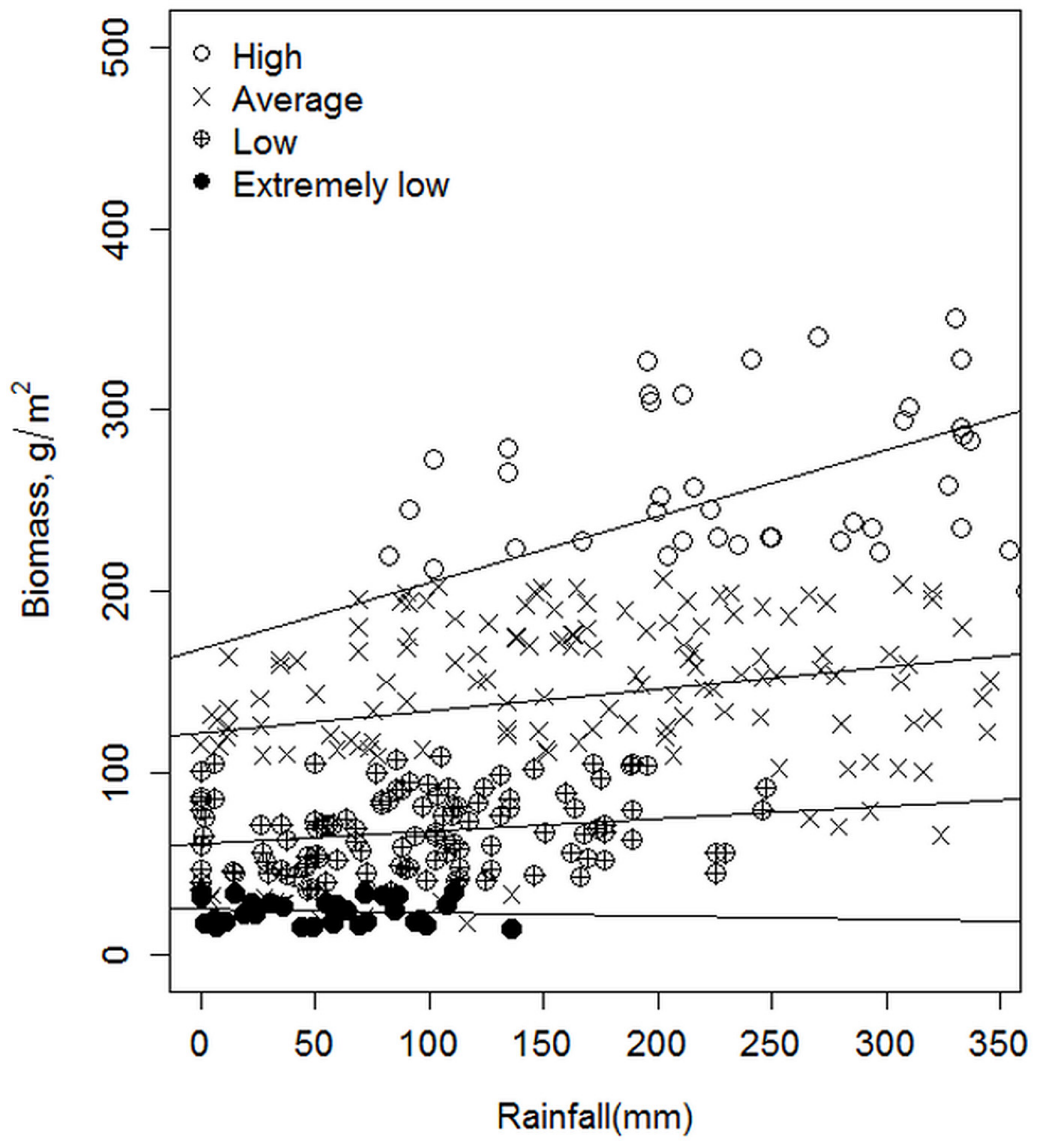
The grass biomass produced per unit of rainfall classified by high, average, low and extremely low biomass deviations from the mean. The growth for a given level of rainfall reflects the starting biomass level. Periods of low and extremely low biomass produce far less growth for the same level of rainfall compared to average and high biomass periods. An Analysis of Covariance (ANCOVA) to compare the four regression lines above shows that their slopes are significantly different (F = 7.01, p = 0.0001).

We modeled the pasture data using the generalized least square (gls) function in the nonlinear mixed effects (nlme) library [46] of R v3.03 [47]. The function fits regression models with a variety of correlated-error and non-constant error-variance structures, hence accounting for possible heteroskedasticity [48, 49]. Since the time series grass biomass data was also autocorrelated (DW = 0.3208, p < 0.0001), this modeling method was appropriate. We further investigated multicollinearity between possible predictor variables in the model using variance inflation factor (VIF) [50]. We utilized the *vif* function in R package VIF [51]. The function selects variables for building a linear model based on the variance inflation factor. Antecedent rainfall, NDVI and grazing pressure were all selected to be included in the model with grass biomass as the response variable. The analysis was done at four levels. First, we considered the overall mean monthly grass biomass (g/m^2^) as a response variable and tested the effect of three predictor variables: rainfall, NDVI and herbivore off-take, measured by the percentage of pasture grazed. Second, we stratified the plot data into four periods of high, average, low and extremely low biomass levels, based on standard deviations of a given month from the long-term mean. High biomass months were classified as those falling one standard deviation or more above the mean, average months as those falling within three-quarters standard deviation either side of the mean, low biomass months as those falling between three quarters and one standard deviation from the mean, and extremely low months as those falling one standard deviation or more below the mean (Fig 3). We then looked at the relationship between each of the predictor variables and the level of biomass deviation from the mean. Third, we divided the response variable into green and dry grass biomass and reran the analysis. Finally, we looked at the influence of habitat on drought indices by classifying the 20 plots into the four major habitats in Amboseli: bushlands, plains, woodlands and swamps [52], and reran the analysis for each separately.

We first considered the model:

y=Xβ+ε (1)

where *y* is a column vector representing time series of grass biomass data collected for over 28 years from the 20 permanent plots (Fig 1), *X* represents a matrix whose columns are possible predictors of grass biomass, in this case rainfall, NDVI and herbivore off-take (grazing pressure). *β* is the parameter column vector. The error vector is given as *ɛ*. In this model, the variance is *var* *ɛ* = *σ* ^2^∑, where *σ* ^2^ is unknown and ∑ is estimated from the data by using the ***gls*** *function* in the *nlme library* of R v3.03. The grass biomass data exhibited an autoregressive process of order one (DW = 0.3208, p < 0.0001).

The parameter estimates are obtained by solving

$$\hat{\beta }={\left(X^{T}\sum^{-1}X\right)}^{-1}X^{T}\sum^{-1}y$$ (2)

The parameter variance is given by:

$$var\hat{\beta }={\left(X^{T}\sum^{-1}X\right)}^{-1}\sigma^{2}$$ (3)

Details of the formulations are given in the Appendix A.I.

We tested the variations in plant biomass, rainfall, NDVI and grazing pressure to determine if the long-term data is stochastic or shows any significant periodicity. We used spectral analysis to identify periodicity in the proxies and grass biomass for the Amboseli area.

To investigate biomass trends, persistence effects and causes, we fitted a power model given by:

$$D=\kappa G^{\alpha }\;\;\kappa >0,\;\alpha \neq 0$$ (4)

showing biomass per unit of rainfall *D*(gm^−2^/*mm*) as a function of grazing pressure (*G*). Calculation of the variance explained (R^2^) was made after model linearization [53].

A logistic regression model [54] was used to calculate the probability of a significant cycle in proportion to the biomass measured for all 20 permanent sampling plots. High biomass habitats such as the swamp are more likely to experience a significant cycle compared to low biomass habitats.

## Results

### Patterns of biomass fluctuation and the reliability of proxy measures

Fig 2 shows that grass biomass averaged for all monitoring plots over the study period is highly seasonal, corresponding to the bimodal rainfall patterns in Amboseli. The results also show large non-seasonal fluctuations in biomass, strong persistence effects and a steady decline in biomass from the 1980s onwards that does not obviously correspond to rainfall patterns or NDVI, but does correspond to an increasing grazing pressure. Table 1 shows the results of rainfall, NDVI and grazing pressure as measures of plant biomass fluctuations, based on a generalized least square model (gls). All proxy measures are significantly correlated with total plant biomass over the study period and are therefore useful proxies. However, the reliability of rainfall and NDVI as proxies declines with increasing biomass deficit from the mean and fall to insignificance at extremely low biomass periods. The exception is grazing pressure, which is highly significant in low biomass periods (t = 3.679, p = 0.0009). In the case of green biomass, all proxy measures are significant over the study period and for high biomass periods. All proxies decline to insignificance during extreme deficits, except for NDVI, which remains marginally significant as a measure of green biomass (t = 1.983, p = 0.057). In the case of dry biomass, which predominates in the dry season, NDVI is insignificant for all periods, rainfall is significant for all but extremely dry periods and grazing pressure is significant for all periods, including extremely low (t = 3.193, p = 0.001).

**Table 1:** The reliability of rainfall and NDVI as proxy measures of biomass shortfall declines in periods of pasture deficit to insignificant levels. In contrast, the reliability of grazing pressure (percentage grazed) as an indicator of biomass deficit increases in significance with shortfall.

Response Variable	Period	Proxy index	*β*	SE(*β*)	t-value	p-value
Total biomass	All-time	Percentage Grazed	-0.323	0.099	-3.248	0.0010***
		Antecedent Rainfall	0.205	0.038	5.398	< 0.0001***
		NDVI	1.543	0.126	12.208	< 0.0001***
	High	Percentage Grazed	1.397	0.380	3.672	0.0010***
		Antecedent Rainfall	0.239	0.073	3.289	0.0200*
		NDVI	2.115	0.261	8.095	< 0.0001***
	Average	Percentage Grazed	1.397	0.380	3.672	0.0005***
		Antecedent Rainfall	0.239	0.073	3.289	0.0170*
		NDVI	2.115	0.261	8.095	< 0.0001***
	Low	Percentage Grazed	-0.378	0.155	-2.440	0.0160***
		Antecedent Rainfall	0.205	0.034	5.961	< 0.0001***
		NDVI	0.613	0.159	3.852	0.0001***
	Extremely low	Percentage Grazed	0.265	0.072	3.679	0.0009***
		Antecedent Rainfall	0.018	0.029	0.609	0.5470
		NDVI	-0.008	0.111	-0.061	0.9464
Green biomass	All-time	Percentage Grazed	-0.615	0.099	-6.240	< 0.0001***
		Antecedent Rainfall	0.168	0.040	4.191	< 0.0001***
		NDVI	1.515	0.129	11.747	< 0.0001***
	High	Percentage Grazed	-0.131	0.064	-2.028	0.0450*
		Antecedent Rainfall	0.097	0.032	3.017	0.0030**
		NDVI	0.686	0.100	6.884	0.0001***
	Average	Percentage Grazed	-0.270	0.441	-0.612	0.5430
		Antecedent Rainfall	0.142	0.086	1.650	0.1050
		NDVI	2.238	0.313	7.159	< 0.0001***
	Low	Percentage Grazed	-0.182	0.136	-1.343	0.1820
		Antecedent Rainfall	0.182	0.042	4.319	< 0.0001***
		NDVI	1.134	0.145	7.815	< 0.0001***
	Extremely low	Percentage Grazed	0.010	0.065	0.161	0.8730
		Antecedent Rainfall	-0.024	0.026	-0.934	0.3580
		NDVI	0.217	0.109	1.983	0.0570
Dry biomass	All-time	Percentage Grazed	0.319	0.044	7.292	< 0.0001***
		Antecedent Rainfall	0.093	0.018	5.251	< 0.0001***
		NDVI	-0.012	0.058	-0.210	0.8340
	High	Percentage Grazed	0.317	0.028	11.382	< 0.0001***
		Antecedent Rainfall	0.029	0.014	2.112	0.0370*
		NDVI	0.007	0.041	0.177	0.8600
	Average	Percentage Grazed	1.487	0.249	5.963	< 0.0001***
		Antecedent Rainfall	0.121	0.043	2.824	0.0070***
		NDVI	-0.185	0.160	-1.163	0.2500
	Low	Percentage Grazed	0.494	0.058	8.521	< 0.0001***
		Antecedent Rainfall	0.101	0.019	5.310	< 0.0001***
		NDVI	-0.064	0.063	-1.006	0.3160
	Extremely low	Percentage Grazed	0.138	0.035	3.913	0.0010***
		Antecedent Rainfall	0.005	0.013	0.406	0.6880
		NDVI	0.028	0.058	0.472	0.6410

sig: ‘***’,0.001 ‘**’,0.01, ‘*’ 0.05

### Habitat variations in biomass and proxy measures

**Fig 4:**
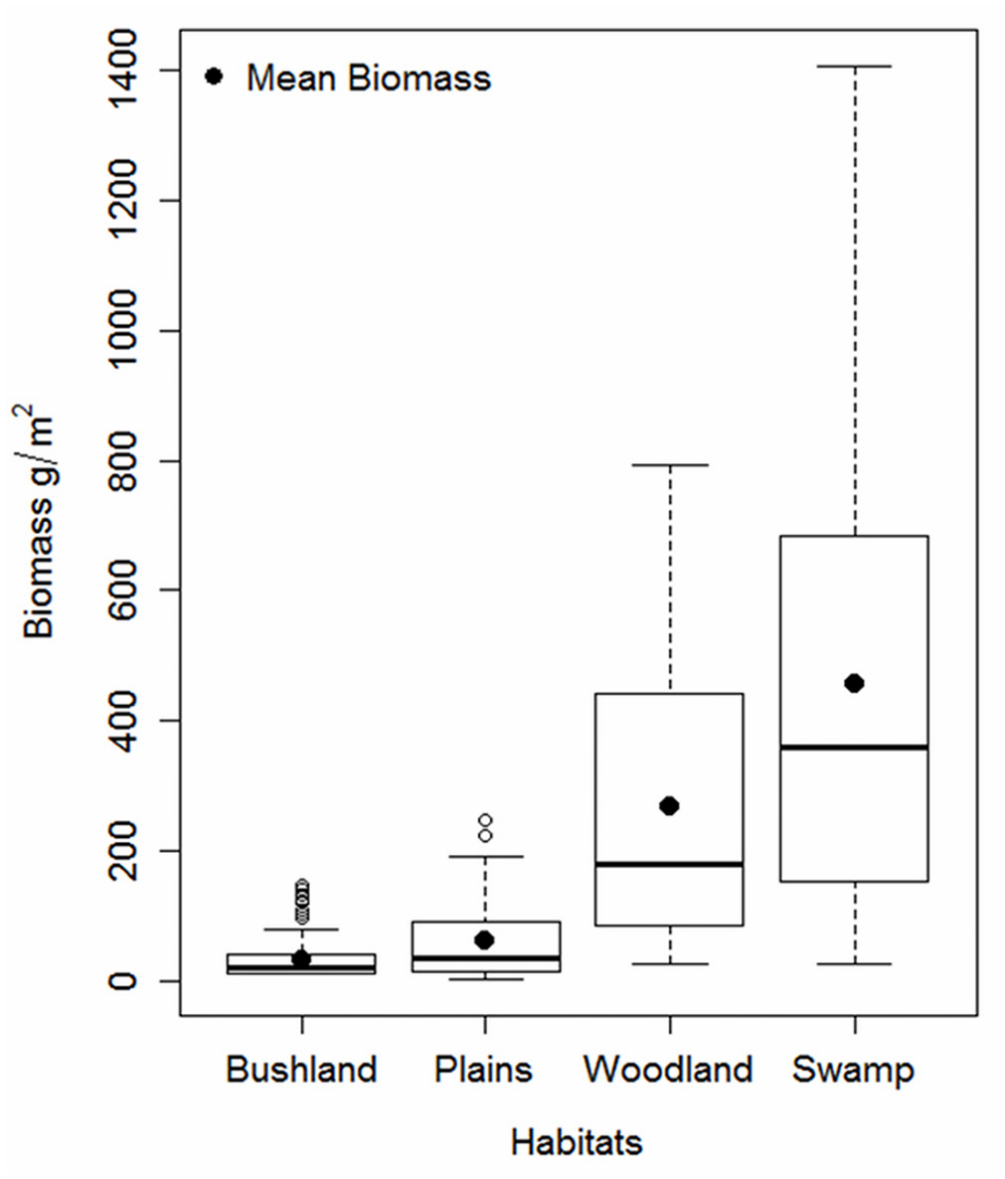
The variance in pasture biomass increases in proportion to the mean biomass in a habitat. Large herbivores of the Amboseli ecosystems move from low to high biomass areas in the course of a season. The large variance in high biomass habitats largely reflects more occasional use than the use of regular low biomass habitats.

Habitat differences in the seasonality of plant growth and senescence influence herbivore migrations and the survival of large ungulates in droughts. In Amboseli the permanent swamps and woodlands draw on aquifer flows from Kilimanjaro and are more productive and less seasonal than the rain-fed bushlands and plains habitats [55]. Fig 4 shows that plant biomass varies greatly between the four major habitats, all of which fall within the same local rainfall regime. Biomass variance is small within habitats, large between habitats (F = 93.96, p<0.0001) and increases in proportion to total biomass.

The reliability of rainfall and NDVI as proxies of plant biomass during periods of extreme shortfall, falls along the biomass gradient from bushlands, plains and woodlands to swamps (Table 2). Neither proxy is significantly correlated with woodland or swamp biomass. Grazing pressure, in contrast, is significantly correlated with plant biomass in all habitats, including the woodlands and swamps. The biomass gradient across habitats corresponds to herbivore movements between habitats as the dry season intensifies. The woodlands and swamps act as drought refuges during droughts.

**Table 2:** ANOVA table of the generalized least squares (gls) regression results relating proxy indices of pasture abundance to measured biomass during periods of extreme biomass deficit in the four major habitats of Amboseli.

Habitat	Proxy index	df	F-value	p-value
Bushlands	Percentage grazed	1	1299.184	<.0001***
	Antecedent Rainfall	1	7.403	0.0140*
	NDVI	1	14.050	0.0020**
Plains	Percentage grazed	1	66.927	<.0001***
	Antecedent Rainfall	1	6.793	0.0180*
	NDVI	1	7.555	0.0130*
Woodlands	Percentage grazed	1	33.728	<.0001***
	Antecedent Rainfall	1	1.443	0.2450
	NDVI	1	2.026	0.1720
Swamps	Percentage grazed	1	78.134	<.0001***
	Antecedent Rainfall	1	0.249	0.6240
	NDVI	1	0.117	0.7360

sig: ‘***’,0.001 ‘**’,0.01, ‘*’ 0.05

### Biomass periodicity

**Fig 5:**
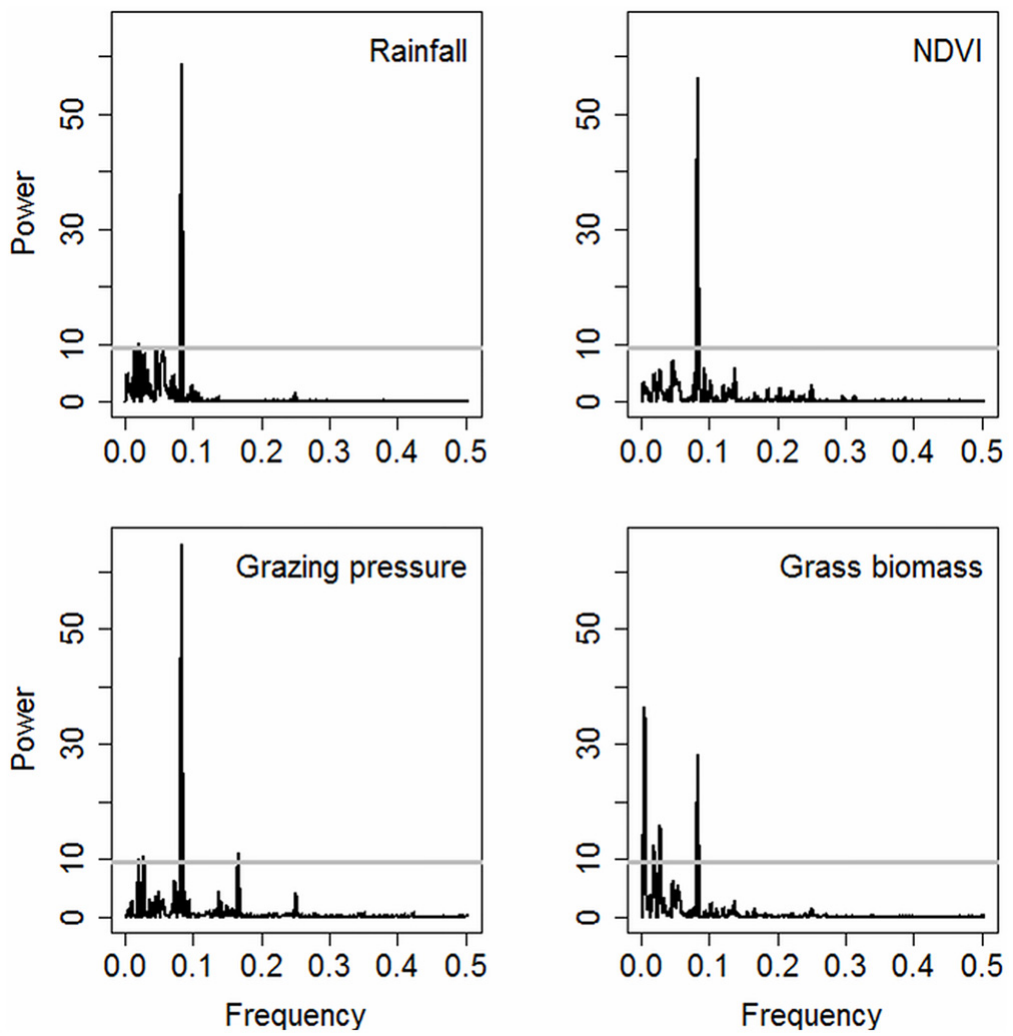
Spectral analysis of proxy indices and measured biomass averaged for all habitats. The frequency peaks above the grey line signify significant 1, 3 and 16-years cycles for grass biomass. The proxies show a significant annual cycles correspond to the seasonal growth cycles.

**Fig 6:**
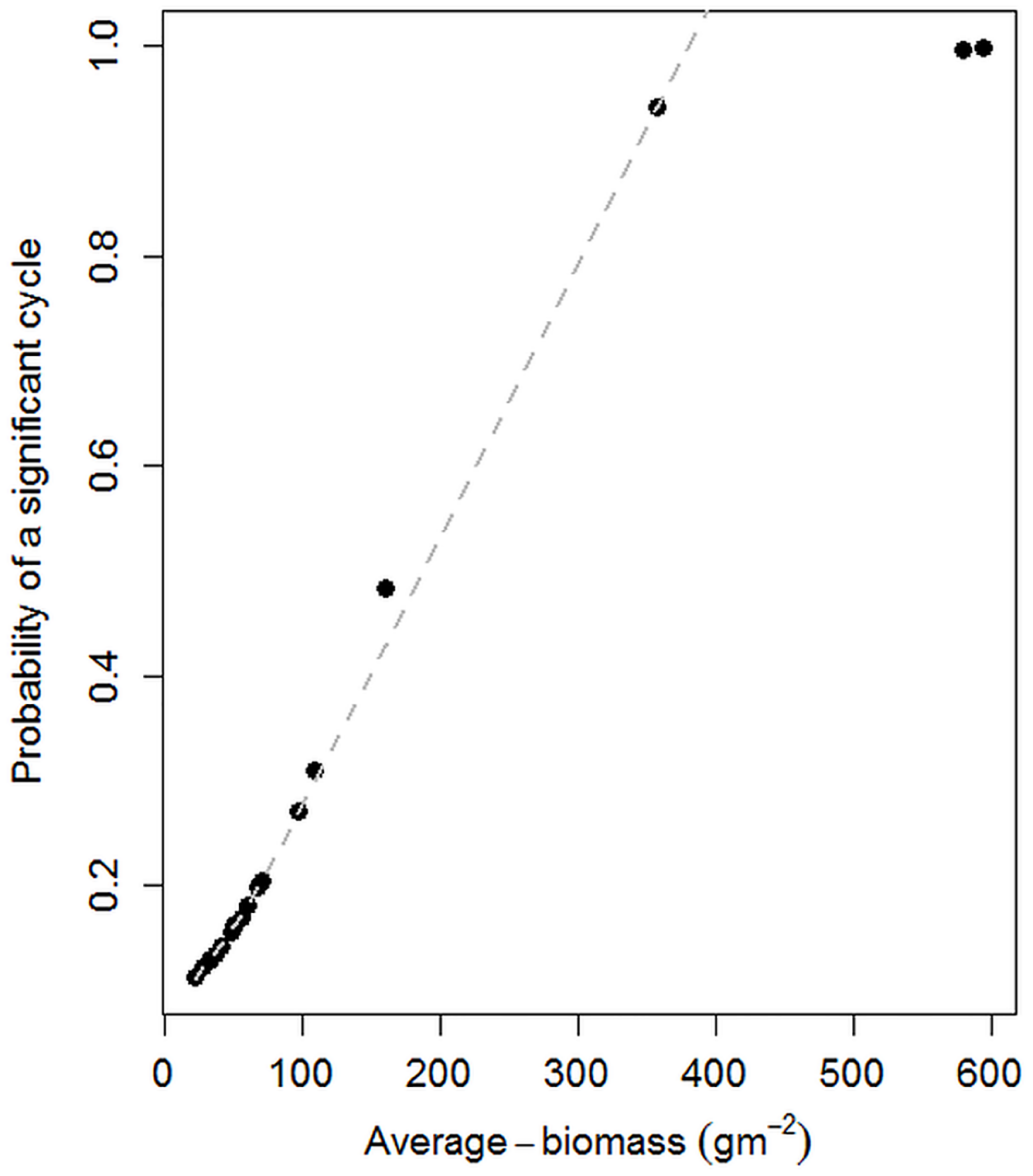
Average plant biomass and the probability of significant cycles plotted for the 20 permanent pasture plots monitored since 1980s. The probability of cycles increases in proportion to average biomass.

The frequency of the strongest peak (power) for rainfall was 0.08321 cycles per month, which corresponds to 12 months (annual) per cycle. For NDVI, 0.08285 cycles per month again corresponding to a one-year cycle. The same annual cycle applies to grazing pressure (0.08321 cycles per month). However, there were more significant cycles for grass biomass (frequency peaks above the gray line, p<0.05 significance level in Fig 5). The frequency of the strongest peak for grass biomass was at 0.005042 cycles per month, corresponding to approximately a sixteen-year cycle. The second strongest peak was at 0.027378 cycles per month, corresponding to a three-year cycle. The third significant peak was at 0.082853 corresponding to the expected annual grass biomass cycles. (See Appendix A.II for details on calculating cycles). Vegetation biomass averaged over all habitats showed significant 1-year, 3-year, 4 and 16-year cycles (p<0.0001). The short-term biomass cycles correspond to the bimodal rainfall regime seasonal growth cycles. The longer cycles do not correspond to rainfall oscillations, which show no significant long term cycle (Figs 2 and 5). The annual cycles occurred in all habitats (p<0.0001). Longer term cycles varied across habitats, with the longest being a 16-year cycle in the swamps (p<0.0001). High biomass habitats such as the swamp were more likely to experience a significant cycle than low biomass habitats according to the logistic model (Fig 6).

### Biomass trends, persistence effects and causes

**Fig 7:**
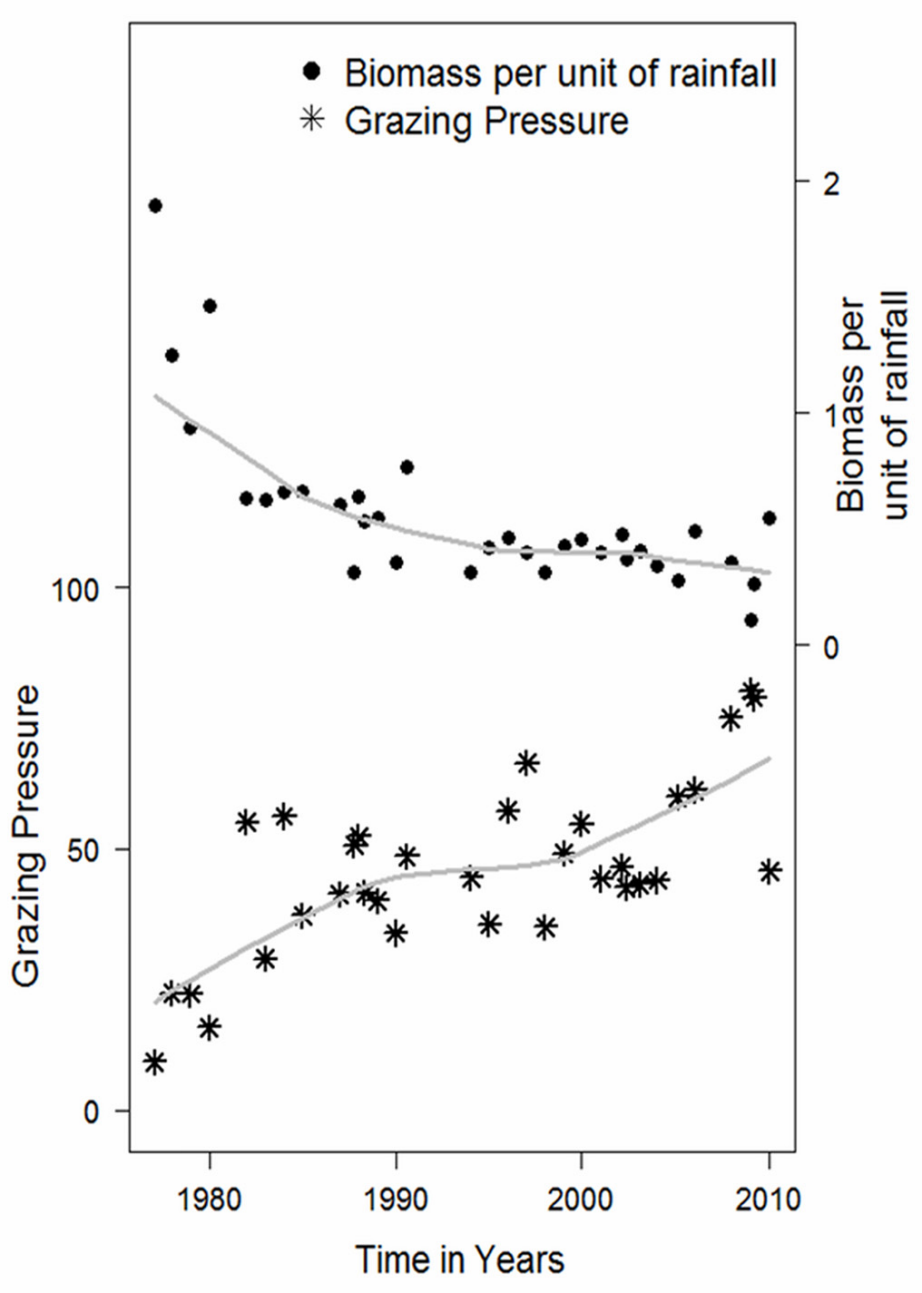
Biomass per unit of rainfall declined significantly over time with rising grazing pressure (t = -3.184, p = 0.0032).

Monthly plant biomass measured at the 20 ground plots declined significantly from 1982 to 2010 (t = -2.346, p = 0.0253). Neither rainfall nor NDVI at peak seasonal growth showed any significant trend over time (Mann Kendall *τ* = 0.148, p = 0.268 and *τ* = 0.014, p = 0.722 respectively) that corresponds to the decline. Grazing pressure, however, showed a strong and significant increase (t = 5.319, p< 0.0001) (Fig 7). The decline in plant biomass is inversely related to the increase in grazing pressure (r = -0.5, p = 0.0032). Further, biomass production per unit of rainfall declined by approximately a third over the study period, corresponding to a doubling in grazing pressure (Fig 7). The parameter estimates from model (4) were highly significant (*κ* = 14.29, p< 0.0001 and *α* = -0.88, p< 0.0001), showing grazing pressure explains 58.5% of the decline in grass biomass per unit of rainfall. The negative value of *α* = -0.88 shows the declining trend.

The persistence effects evident in Fig 2, largely arise from antecedent biomass levels. Fig 3 shows the response of grass to rainfall, classified by standard deviations from the mean into periods of high, average, low and extremely low biomass. High biomass periods produce abundant growth per unit of rainfall, with productivity dropping sharply in periods of low and extremely low biomass. Periods of low biomass will therefore take time to return to higher productivity per unit of rainfall. The probability of extreme pasture shortfalls shows a significant upward trend (Mann Kendall *τ* = 1, p< 0.0008), suggesting that shortfalls are becoming more frequent.

## Discussion

### The reliability of proxies

The results of our study comparing proxy measures of rainfall and NDVI to direct measures of plant biomass across a rangeland ecosystem show that rainfall and NDVI are reliable indicators of green biomass during and after the rains. However, their reliability as indicators falls to insignificance in extended dry periods and for predicting extreme deficits. The reasons are two-fold. First, as green pasture dries, NDVI fails to detect non-photosynthetic plant tissue [56, 57]. Second, residual biomass in long dry periods is determined largely by stocking rates and mobility of herbivores rather than rainfall [58]. The importance of herbivory in determining residual biomass is shown in the highly significant relationship between grazing pressure and plant biomass during periods of extreme deficit (Table 1).

The reliability of proxy measures of pasture biomass also vary with habitat. NDVI and rainfall both predict plant biomass for all periods in the short grass bush and plains habitats used by ungulates during the rains [40]. The significant correlation is due to the rapid offtake of pasture by migrants during the rains, resulting in a short interval between peak pasture growth and the residual biomass in the dry season. The result is a low inter-seasonal variance and low proportion of dry biomass that would otherwise impede the NDVI signature. However, neither NDVI nor rainfall predict biomass in the woodland and swamp habitats where plant growth is driven more by ground water recharge from Mt. Kilimanjaro [34] than by rainfall.

Evidence that the reliability of grazing pressure in predicting biomass rises relative to NDVI and rainfall in proportion to pasture decline (Table 1), and in habitats used as drought refuges (Table 2), gives quantitative evidence to theoretical models of the role of herbivores in determining the state of arid and semi-arid ecosystems [58–60]. Grazing pressure also explains the rising probability of biomass cycles in proportion to plant mass (Fig 6) in the absence of significant rainfall cycles greater than annual bi-modality (Fig 5). Finally, a doubling in grazing pressure also explains the long term decline in biomass (Fig 7) and fall in biomass yield per unit of rainfall.

### Management implications

The rising probability of forage deficits in Amboseli fits pastoral perceptions of a rising frequency of droughts in Kenya [61]. The increasing frequency of droughts has commonly been attributed to climate change [62], but without strong supporting evidence [63]. The results reported here, and studies of the impact of land subdivision [37, 39] point to a rising grazing pressure and sedentarization, coupled with the compression of elephants into the woodlands and swamps of Amboseli due to poaching [64] as the main drivers of the biomass cycles, persistence effects and declining productivity. The results illustrate the role of changing land use practices in grassland deterioration. They also point to the need for better grazing practices that incorporate traditional grazing rotation and reestablish drought refuges as a means of restoring grassland productivity and resilience to drought and climate change [23, 65, 66].

The results of the proxy analysis in Amboseli has a number of implications for monitoring rangelands savanna ecosystems. Direct measures of pasture biomass and systematic monitoring of sample plots using rapid non-destructive methods have several advantages over remote sensing methods. The main advantage is that direct monitoring can be conducted by local personnel engaged in pastoralism. The Amboseli monitoring program has been conducted continuously by David Maitumo, a community member, since 1975. A recent study [67] has shown that such community-based research has a far larger uptake and impact on conservation measures than externally conducted studies. The methods are also simple, cheap and rapidly conducted. Direct monitoring also records a variety of cues such as the ratio of green to dry mass, grass height, cover and species composition, all of which herders use in assessing pasture conditions across their foraging range and in making herding decisions.

An additional advantage of direct monitoring methods is the ease of simultaneously measuring climatic, ecological and socioeconomic variables that determine plant abundance and condition, including the numbers and movements of livestock and wildlife, grazing pressure and trampling impact. Combining the supply side of plant production directly with measures of forage demand and impact gives a more reliable forecast of pasture outlook than supply side measure alone [2, 68, 69]. Direct pasture measurements also have wide ecological applications in assessing primary production, energy flows and phenology [70–72] and in range management applications in calculating stocking rates for sustainable range management [73, 74]. Finally, direct monitoring programs provide ground-truthing data for remote proxy measures and improve the accuracy of remote early warning systems [75–78].

Ground monitoring does have a number of limitations, however. The frequency and scale of monitoring is insufficient to capture the regional scale of pasture abundance and condition at which decisions are made by pastoralist during severe droughts. In the 2009 drought in Kenya, for example, pastoralists moved into Tanzania and Ethiopia in search of pasture. Ecosystem-scale monitoring is insufficient to give a national drought outlook in such circumstances. Here regional proxy measures of rainfall and NDVI complement local ground monitoring programs in drought and famine forecasting [17]. Ground and remote sensing therefore complement each other in scale and accuracy.

Despite its limitations, NDVI has an important role to play in pinpointing late season reserves where residual moisture and greenness provide important drought refuges. These often make up a small fraction of the total migratory ecosystem, but play an inordinate role in buffering herbivore populations from drought [26]. In addition, the effective combination of remote sensing indices with ground based measures of forage production through active community participation will lower the barriers in the uptake of national and regional drought early warning systems in the rangelands.

### Conclusions

We conclude that forage production is not strictly a supply-side, rainfall-dependent variable. Production is affected by animals and people and in turn has a strong bearing on rainfall efficiency via plant physiology, soil infiltration, rainfall capture, and in the longer term, the impact of erosion on plant composition, and soil properties [79]. The results show that endogenous factors play a large role in determining the depth and recurrence of extreme biomass deficits and famines, consistent with earlier studies [80, 81]. Grazing impact has been estimated to account for 35% of all human induced habitat degradation worldwide and 49% in Africa [82]. Better grazing practices are essential in mitigating the impacts of droughts and famines in Africa [83].

## Appendix A.I

The generalised least square (gls) analysis formulation based on [49].

For

y=Xβ+ε (I.1)

The GLS then minimizes

$${(y-X\beta )}^{T}\sum^{-1}(y-X\beta )$$ (I.2)

Which can be solved by:

$$\hat{\beta }={\left(X^{T}\sum^{-1}X\right)}^{-1}X^{T}\sum^{-1}y$$ (I.3)

By Choleski Decomposition where ∑ = *SS* ^*T*^ we have:

$${\left(y-X\beta \right)}^{T}S^{-T}S^{-1}(y-X\beta )={\left(S^{-1}y-S^{-1}X\beta \right)}^{T}(S^{-1}y-S^{-1}X\beta )$$ (I.4)

Here, *S* ^−1^ *X* is being regresses on *S* ^−1^ *y* and so from Eq (I.1), we have:

$$S^{-1}y=S^{-1}X\beta +S^{-1}\varepsilon$$ (I.5)

Which can be written as

$$y^{\prime }=X^{\prime }\beta +\varepsilon^{\prime }$$ (I.6)

The variance of the new errors is:

$$var\;\varepsilon^{\prime }=var\left(S^{-1}\varepsilon \right)=S^{-1}(var\;\varepsilon )S^{-T}=S^{-1}\sigma^{2}SS^{T}S^{-T}=\delta^{2}I$$ (I.7)

We thus find that:

$$var\hat{\beta }={\left(X^{T}\sum^{-1}X\right)}^{-1}\sigma^{2}$$ (I.8)

Where the error-covariance matrix ∑ is estimated from data.

## Appendix A.II

The purpose of spectral analysis is to estimate the (power) strength of periodic components at all possible frequencies. These components are assumed to be sinusoidal, each with a certain amplitude and phase. Power is proportional to amplitude squared. For instance frequency of the strongest peak reported as 0.08321 cycles per month corresponds to (1/0.08321) = 12 months per cycle, i.e. an annual cycle.
